# Barcodes for genomes and applications

**DOI:** 10.1186/1471-2105-9-546

**Published:** 2008-12-17

**Authors:** Fengfeng Zhou, Victor Olman, Ying Xu

**Affiliations:** 1 Department of Biochemistry and Molecular Biology and Institute of Bioinformatics, and BioEnergy Science Center (BESC), University of Georgia, Athens, GA 30602, USA

## Abstract

**Background:**

Each genome has a stable distribution of the combined frequency for each *k*-mer and its reverse complement measured in sequence fragments as short as 1000 bps across the whole genome, for 1<k<6. The collection of these *k*-mer frequency distributions is unique to each genome and termed the genome's *barcode*.

**Results:**

We found that for each genome, the majority of its short sequence fragments have highly similar barcodes while sequence fragments with different barcodes typically correspond to genes that are horizontally transferred or highly expressed. This observation has led to new and more effective ways for addressing two challenging problems: metagenome binning problem and identification of horizontally transferred genes. Our barcode-based metagenome binning algorithm substantially improves the state of the art in terms of both binning accuracies and the scope of applicability. Other attractive properties of genomes barcodes include (a) the barcodes have different and identifiable characteristics for different classes of genomes like prokaryotes, eukaryotes, mitochondria and plastids, and (b) barcodes similarities are generally proportional to the genomes' phylogenetic closeness.

**Conclusion:**

These and other properties of genomes barcodes make them a new and effective tool for studying numerous genome and metagenome analysis problems.

## Background

The challenges being faced in sorting out short genomic fragments generated by metagenome sequencing projects [1] pose a fundamental question: "does each genome have a unique signature imprinted on its short sequence fragments so that fragments from the same genomes in a metagenome can be identified accurately?" A positive answer to this question could have significant implications to many important genome and metagenome analysis problems such as identification of genetic material transferred from other organisms [2] or through virus invasions [3,4], separation of short sequence fragments generated by metagenome sequencing into individual genomes [5] and phylogenetic analyses of genomes [6].

Understanding the intrinsic properties of genome sequences, either general to all or specific to some classes of genomes, has been the focus of many studies in the past two decades. Earlier work includes the discovery of the periodicity property of DNA sequences across both prokaryotic and eukaryotic genomes [7] and the realization that coding sequences follow Markov chain properties [8-10]. Karlin and colleagues have studied various genome properties based on analyses of *k*-mer frequency distributions, and have observed that the *di-nucleotide relative abundance*, a normalized di-mer frequency with respect to the mono-mer frequencies, is generally stable across a genome measured on *50 K *base-pair (bp) fragments [11-13]. They even suggested that such normalized di-mer frequency distributions can possibly serve as signatures of genomes.

In this paper, we present a barcoding scheme for all sequenced genomes, and illustrate a number of interesting and useful properties of the barcodes, which we can take advantage to solve challenging genome analysis problems. We highlight the power of this barcoding scheme through addressing two application problems: metagenome binning problem and identification of horizontally transferred genes.

## Results

### Barcodes and their properties

We have calculated the barcode for each sequenced prokaryotic genome, using the following procedure. For each genome, we partition its sequence into a series of non-overlapping and equal-sized fragments of *M *bps; then for each *k*-mer (1 <*k *< 6 in this study), we calculate the combined frequency of the *k*-mer and its reverse complement within each partitioned fragment. The *barcode *for each genome is a matrix of *N(k) *columns and *genome_length*/*M *rows, with each element representing the frequency of the corresponding *k*-mer within the corresponding sequence fragment, where *N(k) *is the number of unique combined *k*-mers. Note that *N(k) = 4*^k^*/2 *or (4^k ^+ 4^k/2^)/2, depending on whether *k *is odd or even. For example, *N(4) *= 136. The portion of the barcode corresponding to a fragment in a genome is called the fragment's barcode. In this paper, barcodes are calculated using *M *= 1000 and *k *= 4 unless stated otherwise. A discussion on our choices of the *M *and *k *values is given in Additional file 1, where we can also see that the above "equal-sized" requirement is not necessary.

**Figure 1:**
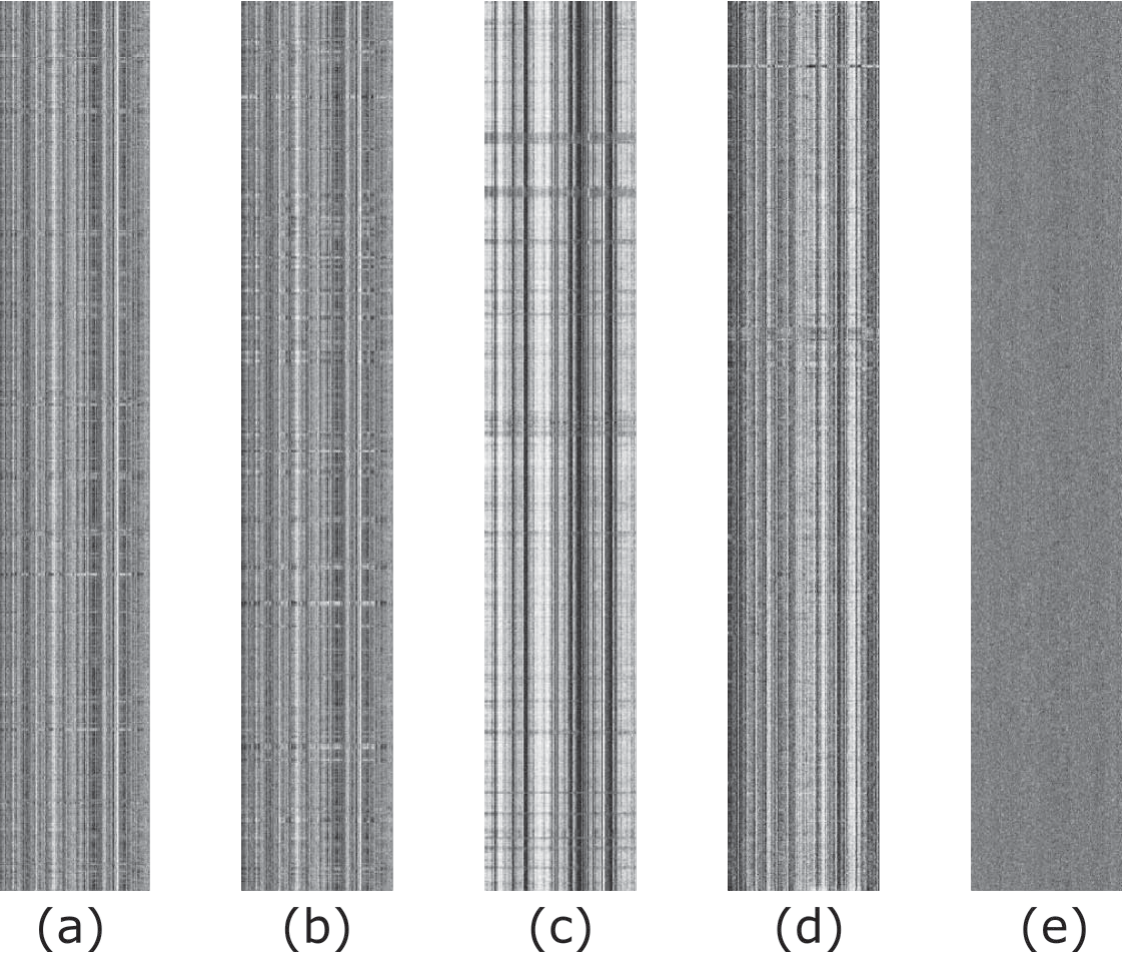
**Barcodes for five prokaryotic genomes**. (a) *E. coli *K-12; (b) *E. coli *O157; (c) chromosome 1 of *B. pseudomallei *K96243; (d) archaean *P. furiosus *DSM 3638; and (e) a random nucleotide sequence generated using a zero^th ^order Markov chain model. The *x*-axis for each barcode is the list of all 4-mers arranged in the alphabetical order, and the *y*-axis is the genome axis with each pixel representing a fragment of *M *bp long.

For each barcode, we have created a grey-level image, a *barcode image*, by mapping the *k*-mer frequencies to grey levels using a procedure given in the METHODS section so that darker grey levels are for lower frequencies. Figure 1 shows the barcode images for five prokaryotic genomes. A key advantage of having barcode images is that they provide an intuitive, informative and global view of genomes, from which various genomic features become immediately apparent. This view can be used to guide our rigorous statistical analyses of genomes. We have calculated the barcode images for all 586 sequenced prokaryotic genomes, which are all accessible at [14], along with the barcode images for other classes of genomes.

**Figure 2:**
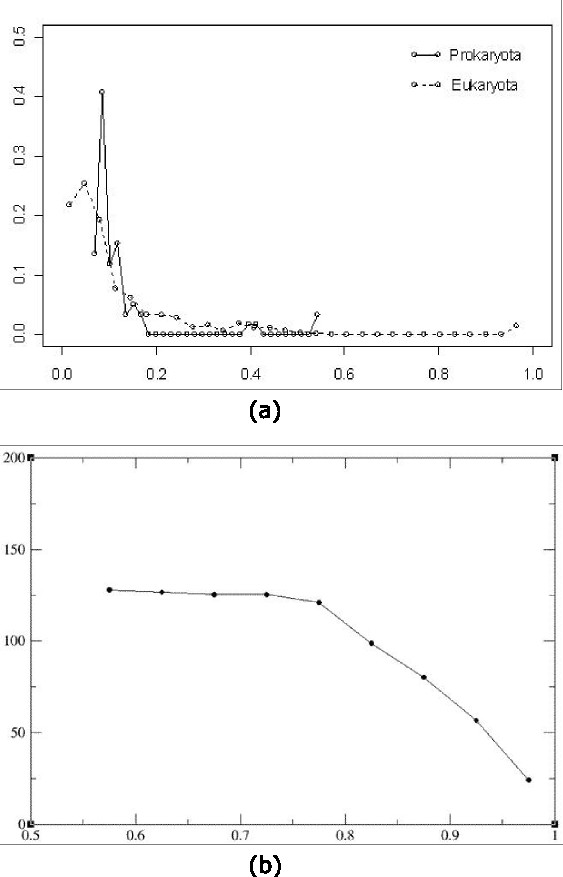
**Basic features of barcodes**. (a) Barcode distance distribution among chromosomes from the same organisms, across all prokaryotic and eukaryotic chromosomal genomes. The *x*-axis is the barcode distance and the *y*-axis is the frequency of chromosome pairs of the same organism having a particular barcode distance. (b) Genome barcode distances *versus *sequence similarities among the corresponding 16S rRNAs (based on the multiple sequence alignment given in DeSantis TZ et al. [26]). The *y*-axis represents the barcode distance, and the *x*-axis is the sequence identity axis between two 16S rRNAs grouped into nine bins, where the sequence identity is calculated as the average sequence identity over all 16S pairs in each bin.

From these barcodes (e.g., Figure 1), we observed that (a) all chromosomal genomes have remarkably stable 4-mer frequency distributions essentially for all 4-mers, giving rise to the vertical bands with consistent grey levels across each barcode; (b) the small fraction of the fragments with clearly different, abnormal, barcodes (horizontal stripes in the barcodes) than the rest of the genome typically represent 2–3 special classes of genes (see discussion later); (c) multiple chromosomes of the same organisms generally have highly similar barcodes (Figure 2(a)) but they each have their unique patterns of abnormal fragments; and (d) the barcodes similarities tend to be generally proportional to the genomes' phylogenetic closeness (Figure 2(b)).

**Figure 3:**
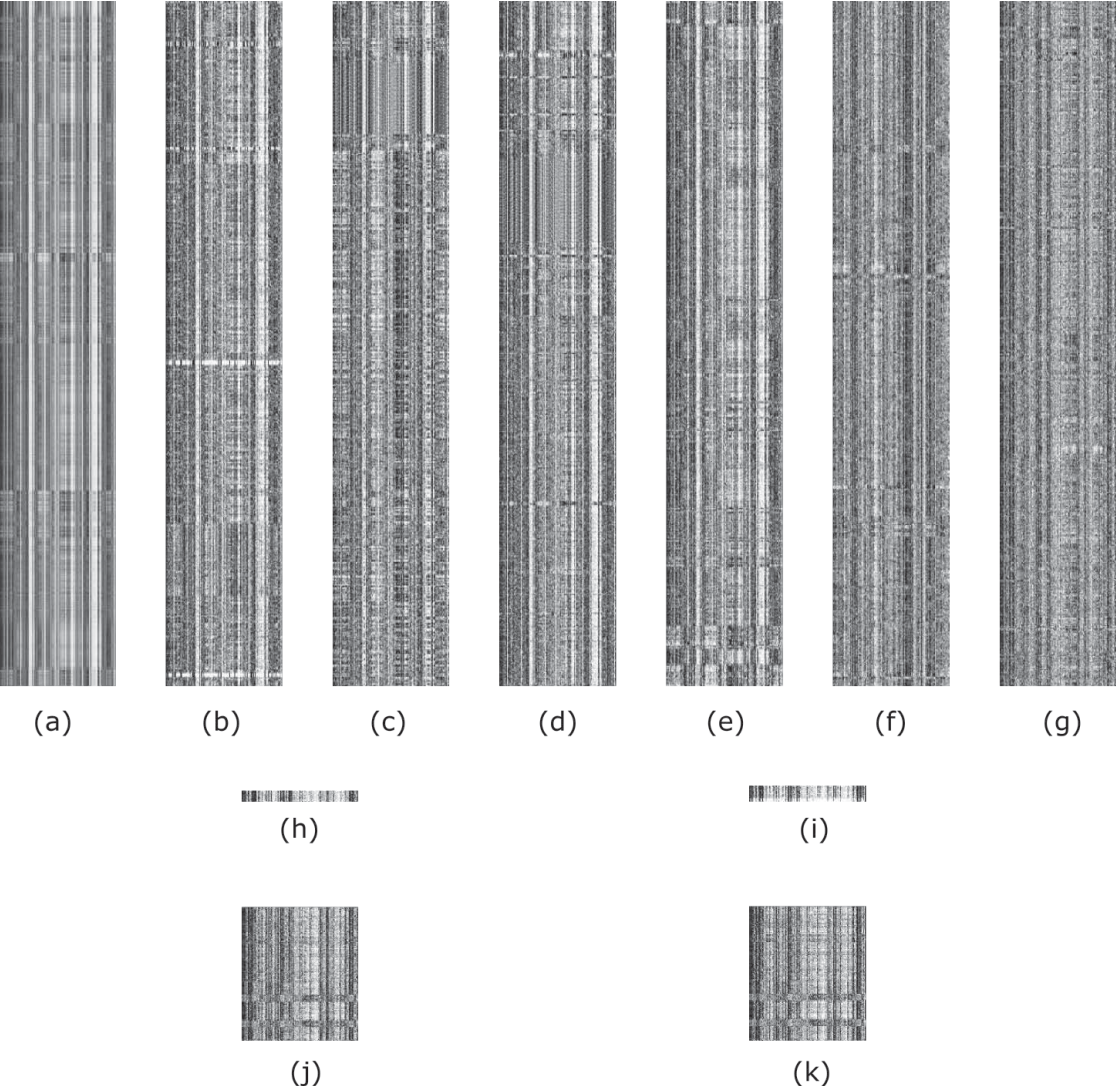
**Barcodes of some organisms**. Barcodes of (a) Human chromosome 1 (226.21 Mbps); major components of human chromosome in a composite form: (b) repetitive sequence, (c) promoter sequence, (d) coding regions and (e) introns; and (f) coding and (g) non-coding regions of *E. coli *K-12. Only a 639-Kbp region of each sequence in (b) – (g) is displayed so each pixel represents the same sequence length. 639 Kbps is used since this is the length of the shortest region among them all, i.e. the total non-coding region of *E. coli *K-12. Mitochondrial genome barcodes of (h) *C. elegans *(13794 bps) and (i) *Drosophila melanogaster *(19517 bps). Plastid genome barcodes of (j) aquatic plant *Ceratophyllum demersum *(156252 bps) and (k) land plant *Populus trichocarpa *(157033 bps).

To understand why a genomic sequence has the barcode property, we have examined random nucleotide sequences generated using different models, including Markov chain models of order from 0 to 6. We observed that barcodes for random nucleotide sequences generated using a third-order Markov chain model are the closest to the barcodes of genomic sequences in terms of their appearances (Additional file 1), and higher order Markov chain models do not seem to add much to this property. Hence we believe that the barcode property of prokaryotic genomes is mainly due to the third-order Markov chain property of the coding sequences in the genomes, which count for 80–90% of a typical prokaryotic genome. It is worth noting that barcodes for coding and non-coding sequences of the same genome are generally different though they share a weakly similar backbone structure while each of these two classes of (composite) regions generally has highly similar barcodes (Figure 3).

### Extension to other genomes

In addition to prokaryotes, we have also calculated the barcodes for the other classes of sequenced genomes, namely eukaryotic, mitochondrial, plastid and plasmid genomes. For eukaryotes, we studied the barcodes for four key components in eukaryotic genomes, namely the (composite) regions of repetitive sequences, promoter sequences (the 1000-bp upstream region from each translation start), coding regions and introns, respectively (Figure 3(b)–(e)). We observed that (i) different regions in a high-level eukaryotic genome (e.g., human) have similar "backbone" structures in their barcodes, and (ii) the barcodes for the four types of regions have increasingly higher complexity, going from repetitive sequences to coding regions to introns and promoter sequences. This is consistent with the belief that introns and promoter sequences are probably the most information rich among the four because of the possibly large numbers of regulatory elements they encode.

The barcodes of the mitochondrial genomes are generally unique compared to the barcodes of the other genomes as they have a distinct overall appearance (e.g., Figure 3(h)–(i)). Their similar appearance may be the result of all mitochondria originating from *Proteobacteria*. The barcodes of all plasmid genomes also tend to have similar characteristics among themselves, possibly due to being under similar selection pressure caused by their frequent transferring among cell cultures. The barcodes of all the plastid genomes are also generally unique compared to the barcodes of the others (e.g., Figure 3(j)–(k)). For example, a majority of them each consist of two dark horizontal bends toward one end in their barcodes along the genome axis, whose corresponding genomic regions consist of RNA genes such as ribosomal RNAs and tRNAs, plus ribosomal proteins. The fuzzier appearance of the plastid barcodes indicates that their *k*-mer frequencies along the genome axis are not as stable as in the other genomes. The overall similar appearances of the plastid barcodes may be due to all originating from the *Cyanobacteria*.

**Figure 4:**
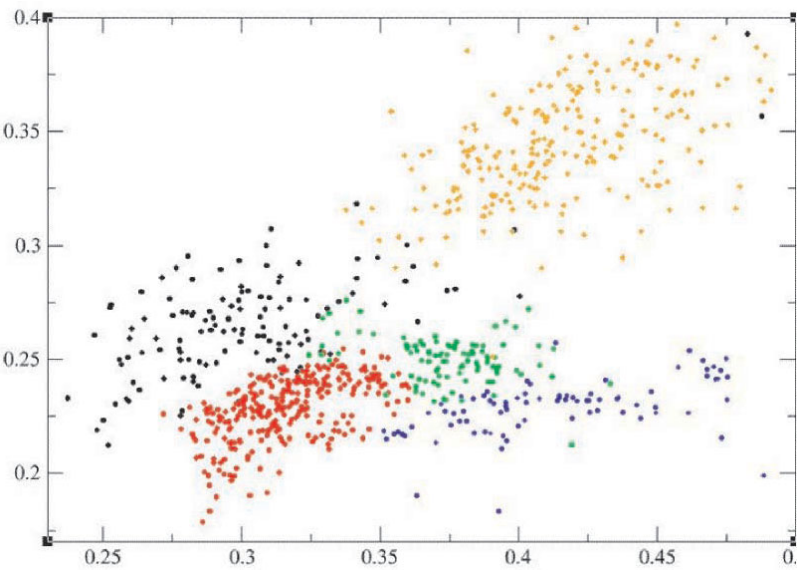
**Barcodes in feature space**. The *x*-axis is the average of variations of the 4-mer frequencies across a whole genome across all 4-mers, and the *y*-axis measures the similarity level among all 1000-bp partitioned fragments of the genome, each represented as a 136-dimensional vector of 4-mer frequencies; Specifically, for each genome, we build a minimum spanning tree [27] based on the 4-mer frequency vectors for its sequence fragments and their distances. The *y*-axis is the averaged weight (distance) of all edges in the minimum spanning tree. The green dots represent prokaryotes (586 genomes), the blue ones for eukaryotes (83 chromosomes), the red ones for plastids (101 genomes with lengths > 20,000 bps), the brown ones for plasmids of prokaryotic genomes (237 plasmids > 20,000 bps) and the black for mitochondria (120 genomes with lengths > 20,000 bps).

One interesting question is "do different classes of genomes have their unique characteristics in their barcodes?" Our answer is yes, based on their highly separable distributions in the feature space defined by two particular features, as shown in Figure 4, one of which measures the overall frequency variation for all 4-mers across the genome's barcode, and the other measures the overall similarity level among all the *M*-bp fragments of the genome, each considered as a vector of 4-mer frequencies. While Figure 2(b) indicates that barcodes generally preserve sequence-level similarities, Figure 4 suggests that barcodes also capture a higher-level similarity beyond individual genome sequence similarities through the textures of their images, which are the common and unique characteristics of different classes of genomes. This property indicates that barcodes are not just a simple visualization tool, instead they have captured some fairly basic information about genomes! From application point of view, we believe that this feature will prove to be useful to metagenome analyses as fragments from different classes of genomes such as eukaryotes, prokaryotes or different organelle genomes, have different characteristics in their barcode images.

### Identification of abnormal sequence fragments

Our procedure for identifying sequence fragments with abnormal barcodes in a genome employs a clustering strategy to divide all the sequence fragments in a genome into two groups: (a) a large group of fragments with their barcodes all similar to each other and (b) the rest (see METHODS section).

Using this procedure, we have identified 30,582 abnormal fragments, covering 30,889 genes across all the complete prokaryotic genomes. Specifically 28,460 such fragments are identified in the 542 bacterial genomes, covering 28,562 genes, and 2,122 such fragments are identified in the 46 archaeal genomes, covering 2,327 genes. We found that the percentage of fragments with abnormal barcodes ranges from 9.40% to 32.32% across all the bacterial genomes, with the average being 12.85%. Among the 46 sequenced archaeal genomes, the percentage of fragments with abnormal barcodes ranges from 9.86% to 23.14%, with the average being 13.58%. Further information can be found from Additional file 1. The detailed frequency information for abnormal fragments across different genomes is in Additional file 2[15].

While we found that it is generally more challenging to study the abnormal fragments in eukaryotes, we did apply the same procedure to different human chromosomes, and found that the percentage of abnormal fragments ranges from 10.08% to 31.32%, with the average being 12.10%.

We have analyzed the abnormal fragments across the prokaryotic genomes, and found the following: ~30% of the abnormal fragments can be explained in terms of (a) horizontal gene transfers, (b) phage invasions and (c) highly expressed genes, based on PHX-PA [16,17] and Prophinder [18], respectively. Among the genes that fall into this 30%, 6.99% are horizontally transferred genes, 4.97% bacteriophage genes and 18.90% highly expressed genes, based on the above two prediction programs – note that these numbers do not add up to exactly 30% since there are overlaps among them. The genes falling into different categories are given in Additional file 3[15]. We have carried out an enrichment analysis of such elements in regions with abnormal *versus *normal barcodes. We found that the highly expressed genes are enriched in the abnormal fragments, with the enrichment ratio > 1 across all the genomes and the average enrichment ratio being 1.90. Similar results hold for the horizontally transferred genes and bacteriophage genes. All the detailed data can be found in Additional file 2

We noted that our estimate of the percentages of "foreign fragments" in bacterial genomes (after deducting the "highly expressed genes") is in general agreement with the previous estimates though different information and techniques are used to derive the estimates [19].

We do not yet have an explanation for the remaining ~70% of abnormal fragments in prokaryotic genomes, although we suspect that they mostly fall into the same three categories – one reason that we could not explain them now is possibly due to the limited coverage of the current databases for horizontally transferred genes, bacteriophage genomes and highly expressed genes. We believe that by using more sophisticated computational procedures, one may be able to derive the level of abnormality of a fragment's barcode in a genome, and possibly link such information to when such fragments were horizontally transferred [20].

### Binning metagenome sequence

The ability to sequence a microbial community has led to the sequencing of at least 7.04 Giga bps of metagenome sequences, already 2.22 times the total complete genome sequences accumulated in the past two decades [21]. These metagenome sequences have opened many doors to new research possibilities, and have posed some challenging problems. One such problem is determining which fragments are from the same organisms in a large pool of metagenomic fragments [22], typically ~1000 bps in lengths after the initial assembly using the Sanger sequencing techniques.

We have applied a clustering algorithm (see METHODS section) for binning sequence fragments together based on their barcode similarities, and tested the clustering strategy on three sets of simulated metagenome data created by cutting actual bacterial genomes into fragments and mixing them together. The three test sets consist of all sequence fragments from three sets of genomes, respectively, extracted from the GenBank. The first set consists of 11 genomes randomly selected from the same genus but from 11 different species (the genus has only 11 sequenced species) while the last two sets each consist of 30 and 100 genomes randomly selected from 30 and 100 different bacterial genera, respectively. The genome names are given in Additional file 4[15].

To assess the binning ability of our algorithm as a function of the fragment size, we have considered fragment size *M *= 1000, 2000, 5000 and 10000. To test the limit of our binning algorithm, we have also considered *M *= 500. For each set of genomes, we partitioned each genome into fragments of size *M*, and then mixed the fragments of the same length into one pool. We then calculated the barcode for each fragment, and did a clustering analysis, assuming that the number of genomes in each pool is known (this information is derivable from the 16S rRNAs). We have carried out binning predictions, one directly on the generated fragments and one on a reduced set of generated fragments, in which we remove 10% of the fragments from each genome whose barcodes are most different from the average barcode of the genome. The consideration is that each bacterial genome has ~13% of fragments with abnormal barcodes on average, which are not expected to be binned correctly with the rest of their host genome. This way we can more accurately assess the binning ability of our algorithm. Table 1 gives the binning results on the three sets of synthetic metagenome data, both the original set and the reduced set.

**Table 1:** Binning accuracies of our barcode-based clustering algorithm.

	11 genomes		30 genomes		100 genomes	
	
	Original genomes	Filtered genomes	Original genomes	Filtered genomes	Original genomes	Filtered genomes
FS = 500 bps	71.10%	77.30%	51. 6%	55.70%	40.50%	41.10%
FS = 1000 bps	79.90%	85.90%	65.30%	70.30%	51.10%	52.60%
FS = 2000 bps	86.30%	91.70%	74.80%	80.60%	61.00%	68.53%
FS = 5000 bps	91.10%	98.10%	86.60%	93.20%	79.40%	81.90%
FS = 10000 bps	95.80%	99.30%	91.90%	97.50%	86.60%	89.18%

The binning accuracy is defined as *(prediction specificity + prediction sensitivity)/2*, and FS is for *f*ragment *s*ize, where both the *specificity *and *sensitivity *are measured in terms of putting the fragments into the correct bin corresponding to each genome, defined by the majority of the fragments in the bin. The column "Original genomes" lists the binning accuracy of our algorithm on all the non-overlapping fragments in each group of genomes, and the column "Filtered genomes" gives the accuracy after removing the 10% fragments with the most abnormal barcodes from each genome.

From the table, we can see that the binning accuracy (into the correct genomes) is high for fragment size *M *= 1000 and above, at both the species and the genus level. From the table, we see that there is a drop in the binning accuracy when the number of the underlying genomes is increased from 30 to 100. This indicates the increased complexity of the problem as a function of the number of underlying genomes.

We have compared our binning performance with the published results by the best available algorithm PhyloPythia [5]. Our comparison indicates that our algorithm gives consistently more accurate and more specific binning results across different fragment sizes. For example, at the species level, our algorithm has better than 50% accuracy on our test set when the fragment size is at least 2000 bps while no binning results at the species level is given in McHardy et al. [5]. At the genus level, the accuracy (the average of binning specificity and sensitivity, extracted from Figure 1(a) and 1(b) in McHardy et al. [5]) by PhyloPythia is 45.5% for fragment size 1000, 56% for 2000, 74% for 5000 and 82.5% for 10000 (no data is provided for 500), all measured in terms of putting fragments into the correct genera while ours is to the correct genomes and with more accurate binning results. It should be noted that the test set used by PhyloPythia is different than ours, which may affect the performance statistics somewhat though we suspect that will be insignificant, considering the sizes of the test sets. Another key difference between the two algorithms is that while PhyloPythia is a supervised learning algorithm, which requires a training set, our algorithm does not require a training set, and hence it is more general.

One thing worth noting is that a prokaryotic genome, on average, has ~13–14% of abnormal sequence fragments, when the fragment size is *M *= 1,000, suggesting that the theoretical limit for binning accuracy should be no better than 86–87%. Similarly we expect that the theoretical limits of binning accuracy for 2,000, 5,000 and 10,000 fragments-based binning should, in general, be no better than 87.36%, 87.58% and 88.4%, respectively.

## Discussion and Conclusion

**Figure 5:**
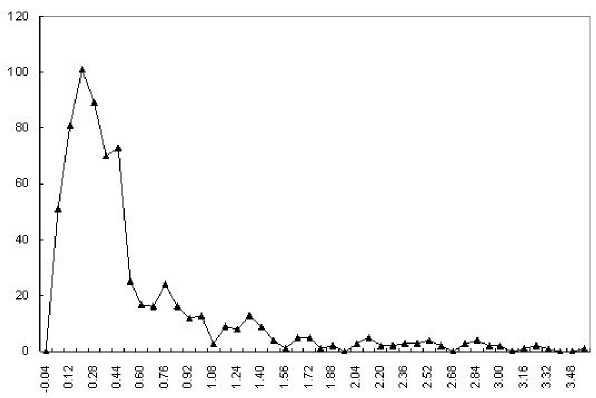
**Distribution of ratios between barcode variations of all prokaryotic genomes and their corresponding randomly generated nucleotide sequences**. For each genome, a *corresponding *random nucleotide sequence is defined as a random sequence of the same length and with the same mono-nucleotide frequencies as those of the genome, generated using a zero^th ^order Markov chain model. The *variation *of a barcode is defined as the standard deviation of the list of the averaged frequencies of all the *k*-mers along the genome. The *x*-axis is the ratio of the barcode variations between a genome and a corresponding random sequence, and the *y*-axis represents the frequency of cases with a particular variation ratio.

A natural question is "do all nucleotide sequences have the barcode property like genome sequences have?" The answer is no, based on the large number of randomly generated sequences that we have examined. Figure 1(e) shows a typical barcode of a random sequence generated using a zero^th ^order Markov chain model. We found that none of the so generated nucleotide sequences has the vertical band structures as in genomes barcodes. More generally, barcodes for genomes and the randomly generated nucleotide sequences have different characteristics as shown in Figure 5.

The barcode analyses in this paper are mainly based on data from prokaryotes. Though we have applied the same barcode model to eukaryotes and made interesting observations, we suspect that the current barcoding scheme is rich enough to capture all the complexity of eukaryotes. Further studies along this direction are clearly needed.

We believe that for many genome analysis problems, particularly for prokaryotic genomes, the barcodes provide a natural, intuitive, information-rich and unified framework for studying them. Further applications of this capability to numerous genome analysis problems can be envisioned, such as phylogeny studies, particularly for genomes without obvious marker genes such as viruses, more thorough examination of different types of genomic regions in eukaryotes, their structures and organization, further studies of horizontal gene transfers, assisting in genome assembly of higher-order organisms (e.g., *populus *which we are currently working on) and possibly many more. We believe that we have only begun exploring the true power of this new capability for genome studies.

## Methods

### Mapping frequencies to grey levels

The frequency of each *k*-mer is mapped to a grey level as follows. We first count the frequency of each k-mer across all prokaryotic genomes, and sort the frequency list S [1:N(k)] in the increasing order of the frequencies with N(k) being the number of k-mers. We then find an integer L, L > 0, and partition S [1:N(k)] into L sub-lists so that the following function is minimized: $\sum_{i=1}^{i=L}\left(S_{i}-\bar{S}\right)$, where ***S***_***i ***_is the sum of all frequencies in the *i*^*th *^sub-list, $\bar{S}$ is the average of *S*, and *L *is a parameter to be determined by the minimization result. For *M *= 1000 and *k *= 4, we found *L *= 14 gives the best value for the above objective function. The computed partition of *S *gives a mapping of frequencies to the grey levels. Note that this mapping is genome-independent so each grey level in the barcodes has the same meaning in different genomes.

### Barcode similarity calculation

We define the *distance *(or dissimilarity) between two barcodes based on their *simplified representations*, each of which is a matrix having the same number of columns of the barcode and the number of grey levels, *L*, used in barcode images as the number of rows; each element in the matrix represents the frequency of the corresponding grey level across each column in the barcode. For two such matrices *M*_1 _and *M*_2 _with *K *columns and *L *rows, we define their barcode distance as

$$\sqrt{\sum_{i=1}^{L}\sum_{j=1}^{K}(M_{1}(i,j)-M_{2}(i,j){)}^{2}}.$$

Clearly this is a generalization of the Euclidean distance between two vectors of the averaged *k*-mer frequencies across each genome, widely used for genome comparisons as in the work of Karlin and colleagues [13,16,23,24] and many others. This is equivalent to the special case of our barcode distance when *L *= 1. Figure S4 in Additional file 1 provides a comparison between the two distances.

### Identification of abnormal fragments in a genome

We have used the following procedure to identify fragments in a genome with substantially different barcodes than the average barcode of the genome. The procedure consists of two key steps. First, for each *k*-mer, we select the fragments in the genome that have the highest or the lowest *X*% of this *k-*mer's frequency among all fragments, with *X *being a parameter. Then we sort all the fragments in the increasing order of the number of times they are selected in the first step, termed function *F(p)*, with *p *being the index of a fragment. Let ***p***_0 _be the fragment index having the highest second-order derivative of *F(p)*. We consider all fragments *p *with *F(p) *> *F(**p**_0_) *to be the non-native fragments of the genome as they have used the most number of *k-*mers with frequencies that are substantially different than the typical *k*-mer frequencies throughout the genome. We found that the abnormal fragment prediction is not very sensitive to the detailed value of *X *within the range from 5 to 20. So we have chosen *X *= 10 as the default value of our program.

The rationale for this procedure is that fragments with higher *F(p) *values represent fragments that have more "abnormal" *k*-mer frequencies compared to the average *k-*mer frequencies in the genome, and hence are more probable to be non-native fragments. By examining the curve of the *F(p) *function, we found that it is convex with one sharp transition point ***p***_0_, indicating a transition point from the typical fragments to the "abnormal" fragments in the genome (see Additional file 1). Hence we have used this point as the separation point between the normal (or native) fragments and the "abnormal"fragments.

### Metagenome binning algorithm

Our binning procedure starts with an application of the CLUMP program [25] to a given pool of fragments (not necessarily of the same lengths) to be clustered based on their barcode similarities. A unique feature of CLUMP is that it is quite accurate in identifying the core elements of each cluster as we have previously demonstrated [25], though a weakness of the algorithm could be that it does not always handle the boundary elements accurately. Hence we have combined CLUMP with a *K*-means based clustering approach that we implemented. After identifying the initial clusters formed by CLUMP based on barcode similarities, assuming that we know the number of clusters to be identified, we pick a seed from each predicted cluster randomly according to the density distribution of the cluster. Then we run the *K*-means algorithm, using the selected seeds. For each pool of fragments, we run this two-step clustering algorithm multiple times, using a different set of seeds for each run. In deciding the number of runs, our rule of thumb based on our experience working on the metagenome data is to use 500 * (the number of clusters),. For each given set of seeds, we run the *K*-means algorithm 10000 iterations. Among all the computed clustering results for each pool, we choose the clustering result ***C***_1_, ***C***_2_,..., ***C***_***K ***_that minimizes the following function as the final binning result:

$$\sum_{i=1}^{K}\sum_{X\in C_{i}}{\left(X-\bar{Xi}\right)}^{2},$$

where ***C***_1_, ***C***_2_,..., ***C***_***K ***_is a partition of a given pool of metagenomic fragments with each *C*_*i *_being a subset of the pool and $\bar{X_{i}}$ being the average of the barcodes of all fragments in ***C***_***i***_, ***i ***= 1,..., ***K***.

## Authors' contributions

Y.X. conceived the project. F.Z. and V.O. analyzed the data and performed the experiments. Y.X. supervised this project as P.I. and wrote the manuscript.

## Supplementary Material

Additional file 1 Supplementary material. Supplementary material 1–3. Click here for file

Additional file 2 Supplementary Table 1. HX is highly expressed gene, HT is horizontally transferred gene, and PH is the phage gene. UnknownGene consists of genes within fragments of abnormal barcodes but do not belong to the above three categories. Click here for file

Additional file 3 Supplementary Table 2. Gene classifications of all the prokaryotic genomes. Click here for file

Additional file 4 Supplementary Table 3. Genomes used in the binning section. Click here for file

## References

[B1] Backhed F, Ley RE, Sonnenburg JL, Peterson DA, Gordon JI (2005). Host-bacterial mutualism in the human intestine. Science.

[B2] Jain R, Rivera MC, Lake JA (1999). Horizontal gene transfer among genomes: the complexity hypothesis. Proceedings of the National Academy of Sciences of the United States of America.

[B3] Frey TK (1997). Neurological aspects of rubella virus infection. Intervirology.

[B4] Rybchin VN, Svarchevsky AN (1999). The plasmid prophage N15: a linear DNA with covalently closed ends. Mol Microbiol.

[B5] McHardy AC, Martin HG, Tsirigos A, Hugenholtz P, Rigoutsos I (2007). Accurate phylogenetic classification of variable-length DNA fragments. Nat Methods.

[B6] Yang E, Bin W, Peng J, Zhang X, Wang J, Yang J, Dong J, Chu Y, Zhang J, Jin Q (2005). Comparative genomics and phylogenetic analysis of S. dysenteriae subgroup. Sci China C Life Sci.

[B7] Trifonov EN, Sussman JL (1980). The pitch of chromatin DNA is reflected in its nucleotide sequence. Proceedings of the National Academy of Sciences of the United States of America.

[B8] Borodovsky M, Sprizhitskii Y, Golovanov E, Aleksandrov A (1986). Statistical patterns in primary structures of functional regions in the E. coli genome. I. Oligonucleotide frequencies analysis. Molecular Biology.

[B9] Borodovsky M, Sprizhitskii Y, Golovanov E, Aleksandrov A (1986). Statistical patterns in primary structures of functional regions in the E. coli genome. II. Non-homogeneous Markov models. Molecular Biology.

[B10] Borodovsky M, Sprizhitskii Y, Golovanov E, Aleksandrov A (1986). Statistical patterns in primary structures of functional regions in the E. coli genome. III. Computer recognition of coding regions. Molecular Biology.

[B11] Karlin S, Burge C (1995). Dinucleotide relative abundance extremes: a genomic signature. Trends Genet.

[B12] Karlin S, Zhu ZY, Karlin KD (1997). The extended environment of mononuclear metal centers in protein structures. Proceedings of the National Academy of Sciences of the United States of America.

[B13] Karlin S, Brocchieri L, Mrazek J, Campbell AM, Spormann AM (1999). A chimeric prokaryotic ancestry of mitochondria and primitive eukaryotes. Proceedings of the National Academy of Sciences of the United States of America.

[B14] Computed_barcodes. http://csbl.bmb.uga.edu/~ffzhou/BoDB/.

[B15] Supplementary_material. http://csbl.bmb.uga.edu/~ffzhou/BoDB/supp/.

[B16] Mrazek J, Bhaya D, Grossman AR, Karlin S (2001). Highly expressed and alien genes of the Synechocystis genome. Nucleic Acids Res.

[B17] Karlin S, Mrazek J (2000). Predicted highly expressed genes of diverse prokaryotic genomes. J Bacteriol.

[B18] Lima-Mendez G, Helden JV, Toussaint A, Leplae R (2008). Prophinder: a computational tool for prophage prediction in pro-karyotic genomes. Bioinformatics.

[B19] Ochman H, Lawrence JG, Groisman EA (2000). Lateral gene transfer and the nature of bacterial innovation. Nature.

[B20] Lawrence JG, Ochman H (1997). Amelioration of bacterial genomes: rates of change and exchange. J Mol Evol.

[B21] Liolios K, Mavromatis K, Tavernarakis N, Kyrpides NC (2008). The Genomes On Line Database (GOLD) in 2007: status of genomic and metagenomic projects and their associated metadata. Nucleic Acids Res.

[B22] McHardy AC, Rigoutsos I (2007). What's in the mix: phylogenetic classification of metagenome sequence samples. Current opinion in microbiology.

[B23] Karlin S, Mrazek J, Ma J, Brocchieri L (2005). Predicted highly expressed genes in archaeal genomes. Proceedings of the National Academy of Sciences of the United States of America.

[B24] Mrazek J, Karlin S (1999). Detecting alien genes in bacterial genomes. Ann N Y Acad Sci.

[B25] Olman V, Mao F, Wu H, Xu Y (2007). Parallel Clustering Algorithm for Large Data Sets with applications in Bioinformatics. IEEE/ACM Transactions on Computational Biology and Bioinformatics.

[B26] DeSantis TZ, Hugenholtz P, Keller K, Brodie EL, Larsen N, Piceno YM, Phan R, Andersen GL (2006). NAST: a multiple sequence alignment server for comparative analysis of 16S rRNA genes. Nucleic Acids Res.

[B27] Cormen TH, Leiserson CE, Rivest RL, Stein C (2001). Introduction to Algorithms.

