# Supplementary material for: Barcodes for genomes and applications
Source: BMC Bioinformatics. 2008 Dec 17;9:546. doi: 10.1186/1471-2105-9-546 (PMC2621371; doi:10.1186/1471-2105-9-546)
Supplement: Additional file 1 — Supplementary material. Supplementary material 1–3. [file 1471-2105-9-546-S1.doc]

# Barcodes for Genomes and Applications:

# Supplementary material

Fengfeng Zhou^*^, Victor Olman^*^, Ying Xu

Department of Biochemistry and Molecular Biology and Institute of Bioinformatics, and BioEnergy Science Center (BESC), University of Georgia, Athens, GA 30602, USA.

*These authors contributed equally to this work.

Correspondence should be addressed to:

Ying Xu

Tel: 001-706-542-9779

Email: [xyn@bmb.uga.edu](mailto:xyn@bmb.uga.edu)

Address: A110, Life Science Building, 120 Green Street, University of Georgia, Athens, GA 30602-7229

Running title:

Barcodes for Genomes and Applications

Keywords:

Barcode, genomics, phylogenomics, metagenomics, binning

**Supplementary Material 1**: *Calculation of genome barcodes using fragment size M and based on k-mers:* There are two factors to consider when selecting the fragment size *M* when calculating the barcode of a genome: (a) the stability of the *k-*mer frequencies across a whole genome, and (b) the desire to deal with short sequence fragments. Clearly the longer the fragment size *M* is for frequency calculation, the more stable the frequencies will be. In the same time, we want to keep the fragment size sufficiently small so we can deal with metagenome data and other genome analysis problems such as identification of fragments transferred from foreign genomes. Our analysis results suggest that *M* = 1000 is a good trade-off between the two competing needs. Figure S1 shows barcodes using different *M* values. By comparing the barcode of (f) and the ones in (a)–(e), we conclude that the “equal-sized” requirement for fragments is not essential when calculating and applying the barcodes.


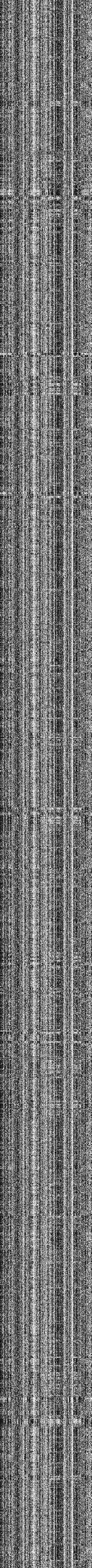

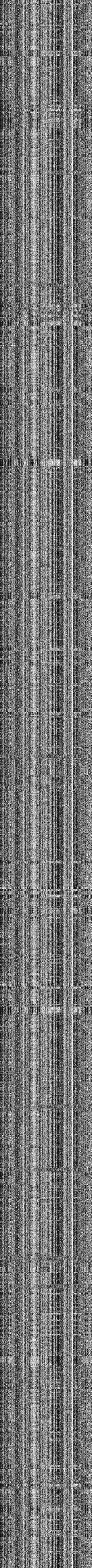

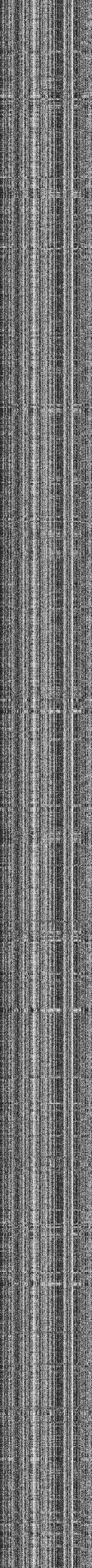

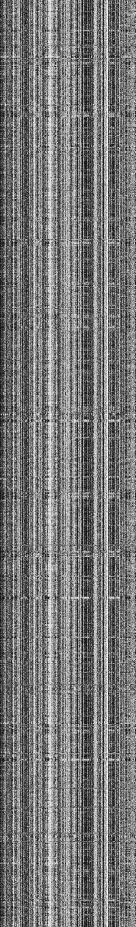

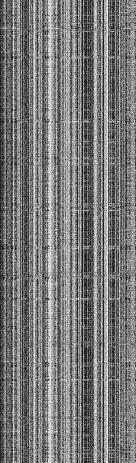

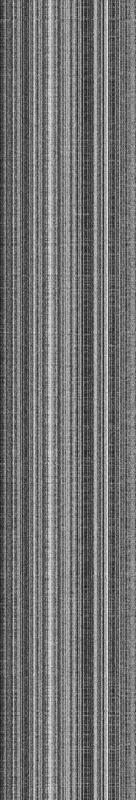


(a) (b) (c) (d) (e) (f)

Figure S1: The barcodes of *E. coli* K-12 based on *M* = (a) 1000, (b) continuation of (a), (c) 2000, (d) 5000 and (e) 10000, respectively. The vertical bands in barcodes have more consistent grey levels (and hence sharper images) in general as *M* increases. (f) The barcode of *E. coli* K-12 using fragments of random sizes that are evenly distributed across the region from 1000 to 10000 bps. Note that each row of pixels represents *M* nucleotides, and hence barcodes based on different *M* values have different heights.

A natural question is which *k* gives a barcode with the highest discerning power among different *k*-mer based barcodes. That is fragments from the same genomes should have in general highly similar barcodes while fragments from different genomes should have distant barcodes. First we need to choose a *k* that the number of unique combined *k*-mers is substantially smaller than the size of sequence fragments, *M*, that we need to deal with, as in the case of metagenomes or identification of foreign genetic material.

Figure S2 gives the frequency (probability) distribution of pair-wise barcode distance between fragments from the same and different bacterial genomes, respectively. Let $F_{k}(d|diff,M)$ and $F_{k}(d|same,M)$ be the total probabilities for the barcode distances to be *< d* for the two cases, respectively. The following quantity gives the lowest total error (errors of type 1 and type 2) when using one single threshold *d* to assign fragments as native and non-native, which we call the *discerning power*.

$D(k,M)=\min_{d>0}(F_{k}(diff,M)+ 1-F_{k}(same,M))\text{ }$.

As can been seen from Figure S3, the function $D(k,M)$achieves its minimum with *k=4* for any size *M* for bacterial genomes.

Figure S2: Probability distributions of barcode distance between fragments from the same and different bacterial genomes.

Figure S3: Discerning power calculated using the formula above on all prokaryotic genomes using an *M =* 1000 (black), 2000 (red), 5000 (green) and 10000 (blue) bps fragment size, respectively. The *x-*axis represents the size of *k*-mers, and the *y*-axis represents the value of *D(k, M)*. The plot suggests that 4-mer based barcodes give the highest discerning power. It also shows how the fragment size affects the discerning power between native and non-native fragments for bacterial genomes.

We have also observed that the combined frequency of *k-*mer and its reverse complement gives a more stable frequency compared to single *k*-mer based barcodes, as illustrated in Table S1. For two random variables (*k-*mers) *X* and Y with variations *σ_X_^2^* and *σ_Y_^2^*, the variation of *(X + Y)/2* is *(σ_X_^2^ + σ_Y_^2^)/4;* and hence when *σ_X_^2^* and *σ_Y_^2^* are approximately the same, and *(σ_X_^2^ + σ_Y_^2^)/4* is about half of *σ_X_^2^* (and *σ_Y_^2^*). For Table S1, we calculated the variations of *k*-mer frequencies, based on 100 randomly selected bacterial genomes, and then tabulated the ratios between the variations of the combined 4mer frequencies and the 4-mer frequencies. This is another reason that we used combined 4-mers instead of individual 4-mers when calculating the barcodes.

| Fragment size | Ratio of combined 4mer/4mer frequency variations |
| --- | --- |
| 1000 bps | 0.7065452 |
| 2000 bps | 0.6958942 |
| 5000 bps | 0.6792713 |
| 10000 bps | 0.6590242 |

Table S1: The ratios of averaged variations of combined 4-mer frequencies and 4-mer frequencies, where the variation of a frequency distribution is calculated, for each genome, as

$$\sum_{i=1}^{N} \sum_{j=1}^{K} (f_{ij}-\overline{f_{j}})^{2}/(KN)$$

where *N* is the number of fragments in a genome and *K* is the number of 4-mers, $f_{ij}$ is the frequency of the *j^th^* 4-mer (or combined 4-mer) in the *i^th^* fragment and $\overline{f_{j}}$is the averaged $j^{th}$4-mer (or combined 4-mer) frequency overall all *i*.

We have compared our barcode distance with the widely used averaged *k*-mer frequency vectors based distance, as shown in Figure S4. The figure indicates that the previous distance is a highly compact and hence a less information-rich version of our distance as each point along the *y*-axis corresponds to a wide range of distance values along the *x*-axis in Figure S4.


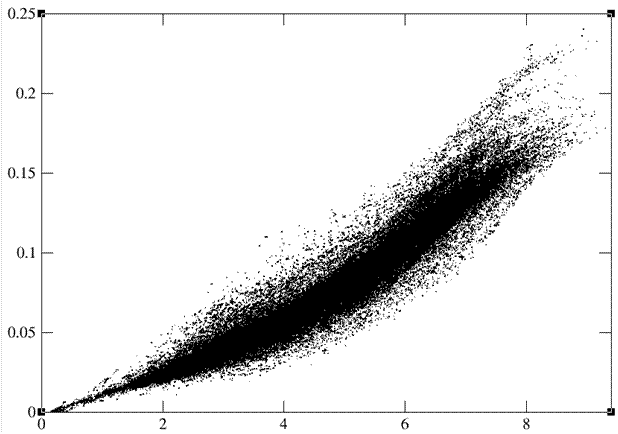


Figure S4: The *x*-axis is our barcode distance and the *y*-axis is the averaged *k*-mer frequency vector based distance mentioned above.

**Supplementary Material 2**: *Comparative analyses of barcodes of randomly generated sequences and genomic sequences:* Figure S6 shows a few barcodes for random nucleotide sequences generated using different orders of Markov chain models. We see that the zero^th^-order Markov chain has no vertical bands structures, and the third-order Markov chain model essentially captures the property of a genome’s barcode.


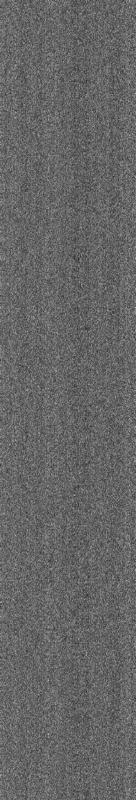

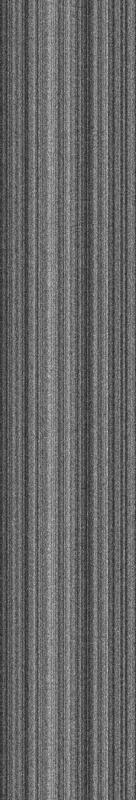

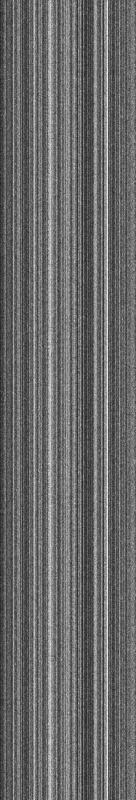

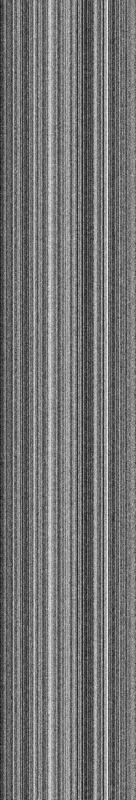


(a) (b) (c) (d)

Figure S5: Barcodes of randomly generated nucleotide sequences. (a) A sequence generated using a zero^th^ order Markov chain model. (b) A sequence generated using a first order Markov chain model. (c) A sequence generated using a third order Markov chain model. (d) A sequence generated using a fifth order Markov chain model. All the Markov chain probabilities used here were calculated based on the genome of *E. coli* K-12.

**Supplementary Material 3:** *identification of abnormal fragments:*

Figure S6: *F(p)* function and the transition point marked as the black dot on the curve of *F(p)* as defined in the METHODS section.

Figure S7: Percentage distributions of abnormal genomic fragments in archaeal and bacterial genomes. The *x*-axis represents the percentage at which level a genome has abnormal fragments, and the *y*-axis is the (normalized) number of occurrences that a genome with a particular percentage of abnormal fragments.
